# Incorporating genetic data improves target trial emulations and informs the use of polygenic scores in randomized controlled trial design

**DOI:** 10.1038/s41588-025-02229-8

**Published:** 2025-06-18

**Authors:** Jakob German, Zhiyu Yang, Sarah Urbut, Pekka Vartiainen, FinnGen, Andrea Gana, Pradeep Natarajan, Elisabetta Patorno, Zoltan Kutalik, Anthony Philippakis, Andrea Ganna

**Affiliations:** 1 Institute for Molecular Medicine Finland (FIMM), University of Helsinki, Helsinki, Finland; 2 Eric and Wendy Schmidt Center, Broad Institute of MIT and Harvard, Cambridge, MA USA; 3 Division of Cardiovascular Medicine, Massachusetts General Hospital, Boston, MA USA; 4 Center for Genomic Medicine Massachusetts General Hospital, Boston, MA USA; 5 Cardiovascular Research Center, Massachusetts General Hospital, Boston, MA USA; 6 Pediatric Research Center, Helsinki University Hospital and University of Helsinki, Helsinki, Finland; 7 Program in Medical and Population Genetics, Broad Institute of MIT and Harvard, Cambridge, MA USA; 8 Personalized Medicine, Mass General Brigham, Boston, MA USA; 9 Department of Medicine, Harvard Medical School, Boston, MA USA; 10 Division of Pharmacoepidemiology and Pharmacoeconomics, Department of Medicine, Brigham and Women’s Hospital and Harvard Medical School, Boston, MA USA; 11 University Center for Primary Care and Public Health, Lausanne, Switzerland; 12 Department of Computational Biology, University of Lausanne, Lausanne, Switzerland; 13 Swiss Institute of Bioinformatics, Lausanne, Switzerland; 14 Broad Institute of MIT and Harvard, Cambridge, MA USA; 15 Analytic and Translational Genetics Unit, Massachusetts General Hospital, Harvard Medical School, Boston, MA USA

**Keywords:** Genetics research, Randomized controlled trials, Epidemiology

## Abstract

Randomized controlled trials (RCTs) remain the gold standard for evaluating medical interventions, yet ethical, practical and financial constraints often necessitate reliance on observational data and trial emulations. This study explores how integrating genetic data can enhance both emulated and traditional trial designs. Using FinnGen (*n* = 425,483), we emulated four major cardiometabolic RCTs and showed how reduced differences in polygenic scores (PGS) between trial arms track improvement in study design. Simulation studies reveal that PGS alone cannot fully adjust for unmeasured confounding. Instead, Mendelian randomization analyses can be used to detect likely confounders. Finally, trial emulations provide a platform to assess and refine PGS implementation for genetic enrichment strategies. By comparing associations of PGS with trial outcomes in the general population and emulated trial cohorts, we highlight the need to validate prognostic enrichment approaches in trial-relevant populations. These results highlight the growing potential of incorporating genetic information to optimize clinical trial design.

## Abstract (web summary)

This study integrates polygenic risk scores with four emulated clinical trials using FinnGen data and shows the feasibility of this approach while highlighting potential pitfalls.

## Main

When they are available, randomized controlled trials (RCTs) are the gold standard to evaluate the comparative efficacy and safety of medical interventions^1^. Randomization ensures that the interventional and noninterventional groups are closely comparable in their characteristics, thus allowing any observed effects to be causally linked to the treatment under investigation. In many real-world scenarios, however, RCT data are not available, and decisions need to be made based on the data at hand.

As the volume of observational data continues to grow exponentially, regulatory bodies such as the U.S. Food and Drug Administration or the European Medicines Agency are increasingly inclined to use real-world evidence (RWE) to gain insights into the effectiveness of medical interventions in clinical practice^2,3^. Trial emulations based on real-world datasets are being increasingly leveraged for this purpose, with ongoing attempts to compare their results with findings from RCTs^4–6^. However, trial emulations can be biased, and traditional epidemiological limitations of observational analyses, including the exchangeability assumption (no unmeasured confounding) remain^7–9^. Residual and unmeasured confounding pose potential threats to the validity of epidemiological studies^10^.

Trial emulations are typically based on claims or registry data that have detailed information on drug prescription and, importantly, purchases, ensuring accurate tracking of patient medication use. These datasets are large but not deep. They do not capture comprehensive biological information, such as genomics and proteomics. Biobank studies, on the contrary, are rich in multi-omics information, but so far, there have been limited efforts to emulate trials within biobanks^11,12^. The main reasons are the small sample size and the difficulty of linking them with claims data, especially in the United States.

Yet, integrating genetic data, alongside comprehensive registry information and expert knowledge, offers a distinctive opportunity to improve trial emulation. For example, genetics offers the opportunity to augment clinical trial design by identifying individuals based on the higher risk of disease (prognostic enrichment) or increased probability of benefit (predictive enrichment)^13^. Further exploration of this concept within a trial emulation setting could pave the way for its implementation in subsequent RCTs. For example, trial emulations can be used to understand if polygenic scores (PGS) can be used for prognostic enrichment within a study population selected with similar inclusion and exclusion criteria as for the RCT, rather than in the general population, as routinely done^14^.

Genetic information is also unique when compared to data available in claims datasets. Genetic information is stable across life; it is not impacted by reverse causation and has low measurement errors. Thousands of genetic variants have been associated with almost every possible measurable human trait, creating a unique catalog of genotype–phenotype relationships. Analogously to the common use of, for example, socioeconomic or behavioral indicators as proxy variables for unmeasured confounders, using PGS as proxy measures for unobserved variables might represent an opportunity to overcome the challenge of accounting for confounding variables that are absent from the dataset^15–17^.

Moreover, genetic differences among treatment groups in an emulated trial could potentially offer insights into residual confounding effects. Using genetic variants as instrumental variables (IVs) in a Mendelian randomization (MR) analysis^18,19^ can help to understand the effect of a potential confounder on the treatment, as well as on the trial outcome at different stages of the emulation process. Genetic information is thus an attractive tool for causal inference and can be used, similar to what has been suggested for other causal inference approaches^17,20^, to identify unmeasured confounding risks.

In this study, we emulate four cardiometabolic RCTs within FinnGen^21^, a Finnish biobank-based study including 425,483 individuals with extensive linkage to drug purchases and other health records data. Leveraging both real data and simulations, we propose new applications of genetics to detect and mitigate confounding risks in trial emulations. Finally, we show how trial emulations within biobanks can inform on the value of PGS for prognostic and predictive enrichment in RCT.

## Results

### Successful emulation of four major cardiometabolic RCTs in FinnGen

We consider four large cardiometabolic RCTs—two (EMPA-REG OUTCOME^22^ (Empareg) and TECOS^23^ (Tecos)) focused on patients with type 2 diabetes (T2D) and two (ARISTOTLE^24^ (Aristotle) and ROCKET-AF^25^ (Rocket)) on patients with atrial fibrillation (AF). Briefly, Empareg established that empagliflozin, an SGLT-2 inhibitor, was associated with a significantly lower risk of cardiovascular events, represented by the composite endpoint three-point major adverse cardiovascular events (3P-MACE). Tecos demonstrated that sitagliptin, a dipeptidyl peptidase-4 (DPP4) inhibitor, was noninferior to usual care for T2D without sitagliptin, with no significant difference in cardiovascular outcomes, as measured by 3P-MACE, thereby confirming the null hypothesis. Aristotle showed that patients with AF at increased risk for stroke using apixaban had a lower risk of stroke or systemic embolism compared to warfarin users. Among a similar patient population, Rocket showed a lower risk of stroke or systemic embolism among those using rivaroxaban versus warfarin.

**Fig. 1:**
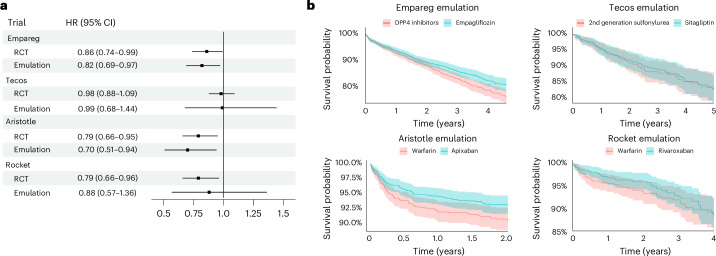
Agreement between RCTs and their RWD emulations in FinnGen. **a**, Comparison of the estimates (HR and 95% CI) of the RCT and their emulation in FinnGen. Points represent estimated HRs; horizontal lines denote 95% CI. The vertical continuous line indicates the null value (HR = 1). Empareg, BI 10773 (Empagliflozin) cardiovascular outcome event trial in patients with T2D mellitus (*n*_RCT_ = 7,020, *n*_Emulation_ = 4,522); Tecos, sitagliptin cardiovascular outcomes study (MK-0431-082; *n*_RCT_ = 14,514, *n*_Emulation_ = 1,094); Aristotle, apixaban for the prevention of stroke in patients with AF (*n*_RCT_ = 18,240, *n*_Emulation_ = 2,292); Rocket, an efficacy and safety study of rivaroxaban with warfarin for the prevention of stroke and noncentral nervous system systemic embolism in patients with nonvalvular AF (*n*_RCT_ = 13,916, *n*_Emulation_ = 1,030). **b**, Kaplan–Meier plots for primary endpoints in FinnGen trial emulations. Lines represent the estimated survival (or event-free) probability over time. Shaded areas indicate 95% CI. Source data

**Extended Data Fig. 1:**
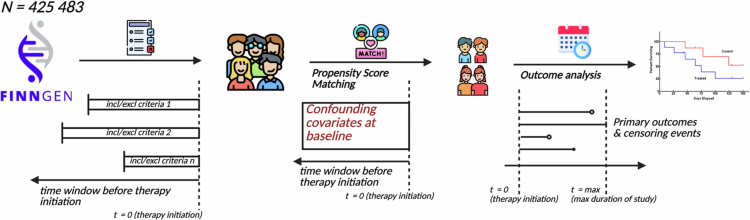
Trial emulation design. This flowchart depicts the involved steps in closely replicating original randomized controlled trials (RCTs) in observational data. Using FinnGen as our data source, we defined the treatment strategies, translated and applied the RCT’s eligibility criteria, selected confounding variables and incorporated 1:1 nearest-neighbor propensity score-matching and performed an outcome analysis after specifying primary endpoints and censoring events, finally obtaining hazard ratios and 95% confidence intervals. The steps are further detailed in the Methods. Incl, inclusion; excl, exclusion; t, time.

We closely replicated these four RCTs in FinnGen, a Finnish biobank study, using the trial emulation framework (Fig. 1) used by the RCT-DUPLICATE initiative^26^, a major trial replication initiative that systematically evaluates the feasibility of using RWE to emulate RCTs and assess the concordance of their findings. Patient characteristics for each trial can be found in Supplementary Table 1. Extended Data Fig. 1 and Supplementary Tables 2 and 3 contain study design and event rate comparisons between the original RCTs and our emulations.

On average, the number of individuals included in the emulated RCTs was smaller than the original trials, with reductions ranging from 36% in the Empareg trial to lower percentages in others, reflecting the large sample sizes typically required for such studies. Despite a considerable number of individuals meeting the inclusion and exclusion criteria, a substantial drop in sample size occurred during 1:1 propensity score (PS) nearest-neighbor matching (for example, *n* = 13,677 eligible individuals in Empareg reduced to *n* = 4,522 after matching). For other trials, the sample size reductions in our emulations approached 90% compared to the original RCTs. Although this resulted in a notable loss of statistical power, the higher event rates observed in our study population helped to mitigate this issue to some extent (Supplementary Table 3).

For all four emulated RCTs, the hazard ratio (HR) estimates were within the 95% confidence interval (CI) of the original RCT’s estimate and aligned with the same direction of the effect. Thus, according to this definition and similar to what was done by the RCT-DUPLICATE initiative, all four trials were ‘successfully’ emulated. However, in Rocket, rivaroxaban was not significantly associated with a lower risk of the composite endpoint stroke/systemic embolism compared to warfarin (HR = 0.88; 95% CI = 0.57–1.36), whereas the original trial observed a significant risk reduction (HR = 0.79; 95% CI = 0.66–0.96).

### PGS differences capture the confounding reduction in emulated trials

Having emulated four RCTs in FinnGen, we assess whether genetic information could be used to evaluate the robustness of the emulation approach with regard to confounding.

As observational data are not randomized, confounding by indication is a major challenge in observational studies of medications. It occurs when the condition that prompts the prescription of a drug is the true cause of the outcome being studied. For instance, doctors may choose a specific drug based on patient characteristics (such as the severity of the disease or potential for adverse reactions), which are not always fully captured in the data. These characteristics can influence the outcome independently of the medication itself. As a result, differences in outcomes between patients on different drugs may be due to underlying differences in patient characteristics rather than the effects of the drugs.

To alleviate this bias, emulated RCTs use a series of precautions, from choosing a sensible comparator group closely mimicking the trial outcome definition to matching individuals for potential confounders^27^. However, not all factors considered when prescribing a drug over a comparator are captured in the data. For example, claims data are often poor in capturing laboratory markers. However, genetic information can be used to proxy, albeit imprecisely, many of these biological traits that are not available in observational data.

**Fig. 2:**
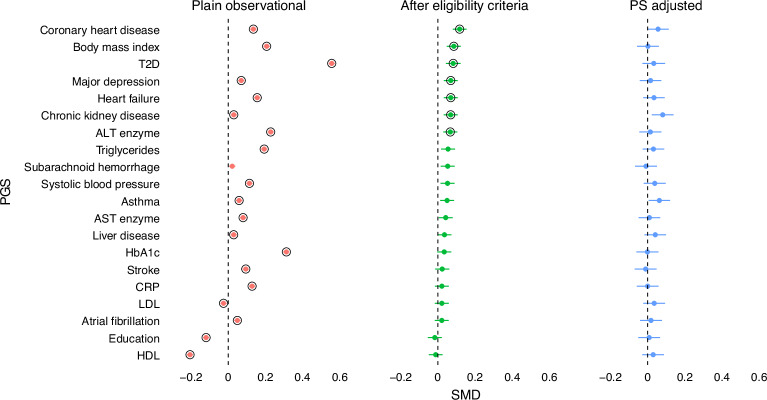
SMD of 20 PGS across different stages of the Empareg trial emulation. Plain observational—empagliflozin initiators versus noninitiators (*n* = 425,483). After eligibility criteria—Empareg trial emulation cohort after applying inclusion/exclusion criteria and including an active-comparator group (DPP4 inhibitor user). The comparison is between empagliflozin initiators versus DDP4 initiators (*n* = 11,349). PS adjusted—Empareg trial emulation cohorts after inclusion/exclusion criteria and a 1:1 PS nearest-neighbor matching for 28 covariates. The comparison is between empagliflozin initiators versus DDP4 initiators (*n* = 4,522). The SDMs of the two trial arms are plotted as point estimates and lines representing their 95% CI. A circle around the point estimates represents statistical significance after a Bonferroni-corrected *P* value threshold (2.5 × 10^−3^). Statistical tests for comparing the means between groups were conducted using two-sided *t* tests, with appropriate adjustments for multiple comparisons via Bonferroni correction. Analogous plots for the other trial emulations can be found in the Supplementary Figs. 1–3. ALT, alanine transaminase; AST, aspartate transaminase; LDL, low-density lipoprotein; HDL, high-density lipoprotein. Source data

With this goal in mind, we computed PGS for 20 traits relevant to cardiometabolic diseases that might capture potential confounders. Some of these traits (for example, coronary heart disease (CHD)) are directly available in the observational data and thus matched upon in the emulated trial; others (for example, C-reactive protein (CRP)) are not available, as FinnGen currently does not contain information on lab measurements. We examined the genetic differences between the trial arms across different stages of the emulation process with the expectation that, by implementing increasing precautions against bias, the differences in genetically inferred factors between the trial arms would reduce. Overall, we observed a decreasing trend in genetic differences, the higher the level of confounder adjustment (see Fig. 2 for Empareg and Supplementary Figs. 1–3 for the other RCTs*)*. In Empareg, we saw a higher imbalance across all PGS in the plain observational setting comparing empagliflozin with noninitiators, which reflects the original RCT design (Empareg versus placebo). We see a particularly high imbalance in the genetically predicted T2D (standardized mean differences (SMD) = 0.56; 95% CI = 0.54–0.57), glycated hemoglobin (HbA1c; SMD = 0.31; 95% CI = 0.30–0.33) and body mass index (BMI; SMD = 0.21; 95% CI = 0.19–0.22), reflecting characteristics of the patient population using empagliflozin. After applying eligibility criteria and considering a sensible comparator group (DPP4 inhibitor users) instead of noninitiators, the PGS differences were overall reduced, but for 7 of 20 PGS remained statistically significant different between the two arms at *P* < 2.5 × 10^−3^, including for CHD (SMD = 0.12; 95% CI = 0.08–0.15) and T2D (SMD = 0.08; 95% CI = 0.04–0.12). Of note, only patients with T2D were included in the RCT emulation stage. Thus, the remaining difference in genetically predicted T2D likely reflects the difference in liability or risk for T2D between the two arms, which can simply be captured by T2D diagnostic codes.

After 1:1 PS nearest-neighbor matching for 26–30 covariates, differences were further reduced, and none was significantly different at *P* < 2.5 × 10^−3^.

For the other three emulated RCTs, we observed similar trends (Supplementary Figs. 1–3). Larger PGS differences in the plain observational analysis were observed for nonactive comparator RCTs (Tecos) versus active-comparator RCTs (Aristotle and Rocket).

### PGS are unlikely to help adjust for confounding in emulated trials

Having established that PGS differences between trial arms track the level of confounder adjustment, one might speculate that directly controlling for PGS in an emulated trial, for example, via PS matching, can help reduce confounders for traits that have not been directly measured.

**Fig. 3:**
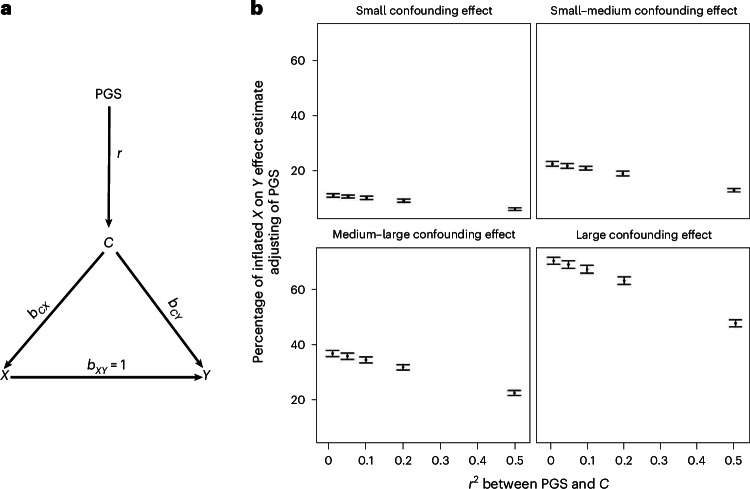
Evaluating the utility of PGS for confounder adjustment. **a**, A DAG illustrates the causal structure between PGS, confounder (*C*), treatment (*X*) and outcome (*Y*). The PGS serves as an imperfect proxy variable for the confounder. The effects of the *C* on the exposure (*X*) and outcome (*Y*) are denoted as *b*_*CX*_ and *b*_*CY*_, respectively. The true unconfounded effect of *X* on *Y* is *b*_*XY*_ = 1. **b**, Simulation study—under this model (Methods), we changed the correlation between *C* and PGS simply by varying *r* and the effect of confounding factor *C* on *X* and *Y* by varying *b*_*CX*_ and *b*_*CY*_. Under each condition, we measured the observed effect of *X* on *Y*, conditioned on PGS and calculated the bias as a percentage of the inflated effect of *X* on *Y*. $\frac{{{b}_{{CX}}}^{2}}{{\mathrm{Var}}(X)}$ = $\frac{{{b}_{{CY}}}^{2}}{{\mathrm{Var}}(Y)}$ = 0.1 for small confounding effect, 0.2 for small-medium confounding effect, 0.3 for medium-large confounding effect and 0.5 for large confounding effect. Under each condition, we carried out experiments for 100 iterations (each *n* = 100,000). Data are presented as mean values and 95% tolerance intervals (±1.96 × s.d.). These simulations show that even if PGS is strongly correlated with the confounder (that is, *r*^2^ = 0.5)—an unlikely scenario, given the correlation between PGS and traits is generally lower—correcting for PGS does not completely account for the bias introduced by the confounder. Source data

To better understand this scenario, we constructed directed acyclic graphs (DAGs)^28^ and performed simulation studies. The DAG in Fig. 3a lays out the graphical relationship between treatment, outcome, confounder and PGS, assuming PGS is directly causal only to the confounder. Similar to other approaches that use proxy measures for unobserved confounding adjustment^17^, if the PGS was a strongly predictive causal instrument for the confounder, one might consider adjusting for PGS when the confounder is not available.

However, several aspects do not support this claim. The first observation is that while PGS is constructed to predict the confounder, it can still be associated with both treatment and/or outcome, independent of the confounder. This is because the PGS is a weighted sum of the effects of multiple genetic variants, some of which can be associated with treatment and/or outcome independently of their effect on the confounder (horizontal pleiotropy). We illustrate this possibility with the DAG and simulations in Supplementary Fig. 4. Thus, controlling for PGS might induce bias by controlling for other nonconfounding factors, including mediators.

The second observation is that PGS are generally weak predictors of traits and diseases^29,30^. Thus, adjusting for PGS would only adjust for part of the variability in the confounders. Under realistic correlation between PGS and the confounder (*r*^2^ between 0.01 and 0.5) and different magnitudes of confounding effect, PGS alone is unlikely to be able to adjust for residual confounding (Fig. 3b and Supplementary Fig. 5).

In addition to the simulations, we empirically tested whether PS matching using PGS could improve trial emulation in the context of the Empareg trial. After applying eligibility criteria, we matched the cohort based on PGS reflecting the covariates of interest. As anticipated, this approach resulted in a significantly unbalanced cohort with respect to the actual phenotypic covariates (SMDs >0.1; Supplementary Table 4). These findings align with the conclusions from our simulations, confirming that PGS are insufficient to control for confounding. Their limited predictive power and potential pleiotropic effects mean that they fail to account for the variability in the corresponding phenotypic traits and may introduce additional bias.

### MR can help identify residual confounders in emulated trials

MR is a powerful method to investigate causal relationships between exposure and outcome variables. By leveraging genetic variants as IVs, MR can help infer causality in observational studies^19,31^.

While MR is typically used to assess the causal relationships between an exposure and an outcome, it can be more generally used as a confounder detector^32^. In this case, genetic variants are used as instruments to test the causal relationships between the potential confounder and both the exposure and the outcome. Unlike PGS, MR selects for variants that are directly associated with the confounder and uses different techniques to limit horizontal pleiotropy (that is, to limit the impact of variants that are associated with the outcome not via the exposure).

**Fig. 4:**
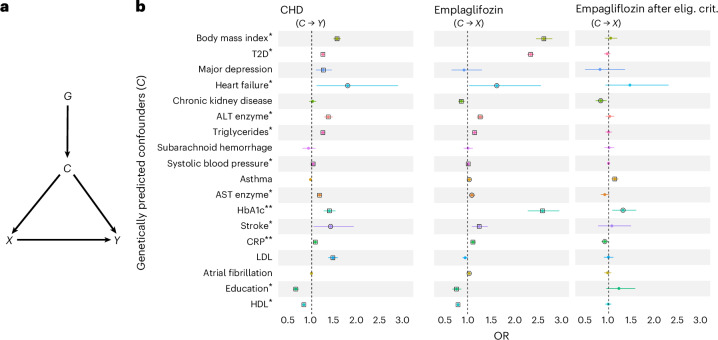
Using MR within the Empareg trial emulation to identify confounders. **a**, A DAG illustrating the relationship between treatment initiation (*X*) and trial outcome (*Y*), as well as the effect of a genetic instrument (*G*) of a confounding variable (*C*) on both the treatment initiation and trial outcome, only through the confounding variable. **b**, Results of an MR analysis using IVW as a statistical test to study the causal effects of 18 traits on CHD, representing the trial outcome, and empagliflozin, representing the treatment initiation. Left: MR for association between 18 traits on coronary artery disease using two-sample MR. Middle: MR for association between 18 traits on empagliflozin initiation in the full study population. Right: MR for association between 18 traits on empagliflozin initiation after applying the RCT’s eligibility criteria. The point estimates represent the ORs with lines representing their 95% CI. For continuous confounders, the OR reflects the change in the outcome variable associated with a 1 s.d. increase in the exposure variable; for binary confounders, the OR represents the change in the outcome variable when comparing the presence versus the absence of the binary exposure. A circle around the point estimates represents statistical significance with a *P* value threshold of 5 × 10^−2^. A square around the point estimate represents statistical significance with a *P* value threshold of 2.8 × 10^−3^. Single asterisk represents putative confounder due to significance in left and middle panels of **b**; double asterisks represent putative confounder due to significance in left and right panels of **b**. Elig. crit., eligibility criteria. Source data

We use an MR framework to better understand whether 19 traits can be considered as confounders in the Empareg emulated RCT. Following the DAG in Fig. 4a, we tested whether the genetic instruments for the potential confounders were associated with both empagliflozin treatment (*G*→*X*) and CHD, a proxy for 3P-MACE (*G*→*Y*).

Two-sample MR studies revealed putative causal effects of 14 of 19 potential confounders on CHD (Fig. 4b, left). There is extensive orthogonal evidence supporting the causal nature of these relationships^33–38^. When performing MR of the confounder on empagliflozin treatment, we observed 15 of 19 traits to have a statistically significant effect (Fig. 4b, middle). Because confounders are defined as variables with an effect on both, the exposure and outcome, we were specifically interested in traits where we observed an effect on both CHD and an empagliflozin treatment. This was the case for 12 traits when emulating Empareg with a plain observational approach. For example, BMI was a likely confounder being putatively causally associated, according to MR, with both empagliflozin treatment (odds ratio (OR) = 2.68 (2.51–2.87), *P* < 2 ×10^−16^) and CHD (OR = 1.55 (1.48–1.64), *P* < 2 × 10^−16^). The putative causal effect on empagliflozin treatment highlights doctors’ tendency to prescribe this medication to patients with higher BMI, a significant risk factor for T2D, which is the primary reason for the drug’s prescription.

After including eligibility criteria and a comparator group (Fig. 4b, right), only two traits, HbA1c and CRP, remain significantly associated, according to MR, with both empagliflozin treatment and CHD.

We further examined whether the causal effects of potential confounders on empagliflozin treatment were mediated through their influence on CHD—that is, whether a physician’s decision to prescribe empagliflozin was guided by the confounder’s impact on the patient’s cardiovascular risk. If that were the case, the confounder could not be defined as such, as it is associated with exposure via the outcome (*C*→*Y*→*X*). We demonstrate that the effects across most putative confounders are small (Supplementary Fig. 6), providing strong evidence that the observed *C*→*Y* causal effect is direct. Additionally, by focusing strictly on the direct effect, we identified educational attainment and aspartate transaminase enzyme as two additional potential confounders.

### Using emulated trials to better evaluate PGS enrichment strategies

PGS can be used to enrich RCTs by identifying individuals based on the higher risk of disease (prognostic enrichment) or an increased probability of benefit (predictive enrichment)^13^. However, to evaluate these potential benefits, it is necessary to test both prognostic and predictive enrichment hypotheses between a study population that is as close as possible to that of the prospective RCT. In fact, PGS have shown different prediction performances across ages, sex, socioeconomic group and comorbidities^14,39^. Moreover, eligibility criteria can restrict the study population to high-risk individuals where PGS might have limited effects^40^.

**Fig. 5:**
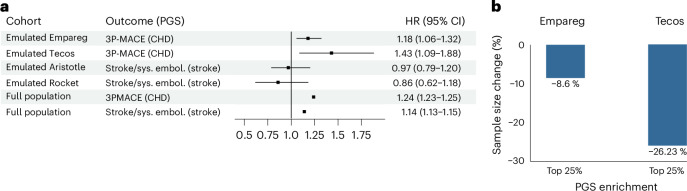
Effect of the PGS on the primary trial outcomes among individuals included in trial emulations and in the full study population. **a**, Effect of the outcome PGS on the primary outcome within each 1:1 nearest-neighbor PS-matched trial cohort using Cox regression and adjusting for the treatment, as well as within the full FinnGen population. HRs per one s.d. increase in genetic liability, and their 95% CIs are illustrated in the central forest plot. Points represent estimated HRs; horizontal lines denote 95% CIs. The vertical continuous line indicates the null value (HR = 1). **b**, Sample size reduction of the emulated Empareg and Tecos trials after enriching the trial cohorts with individuals at the top 25% genetic risk for CHD (top 25% CHD PGS). Sys. embol., systemic embolism. Source data

We first test prognostic enrichment by evaluating whether the PGS for the outcomes of the four emulated trials (that is, CHD and stroke) were associated with the trial outcome within the emulated RCT population (Fig. 5a). We also compare these effects with those observed in the general population to see if the performances of PGS were different when restricting to eligible individuals in the RCTs.

For both Empareg and Tecos emulation, we found the PGS for CHD to be associated with 3P-MACE (HR = 1.18; 95% CI = 1.06–1.32 in Empareg and HR = 1.43; 95% CI = 1.09–1.88 in Tecos). These effects were consistent with what was observed in the full FinnGen (HR = 1.24; 95% CI = 1.23–1.25).

However, for Aristotle and Rocket emulation, the PGS for stroke was not associated with the composite endpoint stroke/systemic embolism (HR = 0.97; 95% CI = 0.79–1.20 for Aristotle and HR = 0.86; 95% CI = 0.62–1.18 for Rocket) despite the significant PGS association in the full population of FinnGen (HR = 1.14; 95% CI = 1.13–1.15).

These results suggest that care should be taken when generalizing the PGS association from the general population to RCT participants.

Given the significant prognostic enrichment for Empareg and Tecos, we calculated the reduction in sample size required to achieve a similar number of events and, consequently, similar statistical power, if we had included individuals in the top 25% of the PGS for CHD. This prognostic enrichment approach strategy would have resulted in −8.6% and −26% reduction in sample size, given all the other inclusion and exclusion criteria being the same (Fig. 5b).

Finally, we tested predictive enrichment in Empareg and Tecos by evaluating the interaction between the PGS for CHD and the treatment arm indicator. A significant interaction would indicate the treatment is more effective in individuals with higher or lower PGS. There was no significant interaction either in the Empareg emulation (*P* = 0.99) or in the Tecos emulation (*P* = 0.24).

## Discussion

In this Article, we show how genetic data can benefit target trial emulation design and analysis and how a trial emulation framework can be used to better understand the value of PGS for RCT design.

To answer these questions, we first emulated four transformative cardiometabolic RCTs in FinnGen using the framework used by the RCT-DUPLICATE initiative^26^. We learned that despite the large sample size and complete national coverage of drug purchases and health outcomes, RCT emulation requires a very large number of individuals. During the PS matching step, on average, 78% of individuals get discarded, as we aimed to match patients as closely as possible to ensure comparability between treatment groups, thus reducing the final sample size. This process led to a reduction in the final sample size by up to 90% compared to the original RCTs. While higher event rates in our study population helped to partially offset the loss of statistical power, the substantial reduction in sample size underscores the need for even larger biobanks to perform such trial emulations effectively. Nonetheless, we were able to successfully emulate all four trials and generate RWE that is concordant with the RCTs’ results. Emulation of smaller RCTs, as, for example, trials for rare diseases, is probably not possible at the current sample size of 500,000 genotyped individuals.

Confounding by indication is particularly severe in observational studies of medications. Our approach leverages the large catalog of genotype–phenotype relationships generated by genome-wide association studies to ‘impute’ biological risk factors that might act as confounders. We show that PGS can be used to identify both measured and unmeasured factors that are unbalanced between the two arms of the trials. Some of these factors are likely confounders; others are not. PGS cannot distinguish between the two. However, PGS can provide a more refined measure of the disease risk than simple disease diagnoses. For example, we show that in the emulated Empareg trial, which includes only patients with T2D, a PGS with T2D was still unbalanced between the two arms of the trial. This might reflect unaccounted confounding by indication based on patients’ T2D risk of T2D-related factors. Reassuringly, we saw that in all emulated trials, the PGS imbalance greatly reduced after PS matching. Our work highlights the importance of matching as a technique for confounding adjustment in observational data and suggests that PGS can be used as an orthogonal assessment of the quality of matching, especially for biological risk factors with genetic bases that are not comprehensively captured by claims or registry data (for example, disease-specific biomarkers). An important limitation of our study was the absence of laboratory measurements. Even when available, laboratory measurements are often inconsistently collected, leading to potential biases. For example, in RCT-DUPLICATE, laboratory measurements were not included as covariates in the PS matching process but instead used in postmatching assessments. While PGS offers a valuable complementary approach to refining confounder assessment, it is not a substitute for direct laboratory data, as it captures only genetic liability and lacks the precision of measured phenotypes. It is also worth highlighting that if a PGS is balanced between trial arms, this does not imply the predicted trait is also balanced. PGS are generally poor predictors of traits, even those with strong genetic bases, and adjusting or matching for PGS, as shown by our simulations and complemented by our empirical analysis, is unlikely to control for the trait they are predicting. One should also consider that if the PGS for a potential confounder correlates with the genetics of the drug response, PGS differences between trial arms should be expected.

Genetics-based IV approaches can, however, be used, together with specialist knowledge and other orthogonal evidence, to identify confounders. While others suggested that MR can be used for confounder detection^41–43^, we applied and extended this framework to emulated RCTs. While MR has numerous limitations that have been extensively described with regard to its most common use to assess the causal relationships between exposure and outcome^19^, here we mention a few limitations that are unique to its use in confounder detection. First, the causal relationship between an exposure (or potential confounder) and a treatment should be interpreted with caution. A putative causal effect is likely to indicate that the exposure is influencing the doctor’s decision to prescribe the treatment and not the treatment effectiveness itself. For example, we identify a negative putative causal effect of chronic kidney disease on empagliflozin treatment, reflecting the former doctor’s decision to avoid prescribing empagliflozin to patients with severely impaired renal function before more recent evidence emerged showing its benefits for these patients^44–47^. Second, the effect of the confounder on the treatment can be mediated by the outcome, or the effect on the outcome can be mediated by the treatment. These effects can be addressed by closely examining the true causal structure and adjusting the confounder effects by the effects of all other pathways, excluding the direct effect of the confounder on treatment or outcome, respectively. Despite these interpretational challenges, MR can be a powerful tool for confounder detection during RCT emulation. At the ‘eligibility criteria’ stage, MR could inform about residual confounding and suggest which factors, if measured, to include in PS matching. After matching, MR can still be used to investigate nonadjusted residual confounding and, together with expert knowledge, better interpret the results of the emulated RCTs.

While we only discussed MR for confounding detection, it would be theoretically possible to use MR for confounding adjustment.

The trial emulation framework is useful to better understand the value of genetics in trial design. The most promising use of trial emulation is to assess the prognostic enrichment for PGS. Individuals enrolled in RCTs are highly selected and do not represent the general population. It would be naive to assume the magnitude of association of a PGS with a certain outcome in the general population would be the same among RCT trial participants. A trial emulation framework can be used to draw a boundary on the expected association between the PGS and the trial outcome, a key piece of information when designing an RCT that uses genetics for either patient selection or as stratification criteria.

A trial emulation is less valuable to understand predictive enrichment because, at the current sample size, biobank-based emulated RCTs still have limited power to test for interaction between PGS and treatment or to stratify individuals in different genetic risk bins.

In conclusion, our work shows that genetic information can improve the design of emulated trials, which, in turn, can help inform the use of genetics in designing RCTs. Some of these results can be extended to other -omics that are getting measured in hundreds of thousands of biobanked samples.

## Methods

### Ethics statement

This study complies with all relevant ethical regulations and was conducted in accordance with the research permits and approvals granted by the respective institutions.

Patients and control participants in FinnGen provided informed written consent for biobank research, based on the Finnish Biobank Act. Alternatively, separate research cohorts, collected prior the Finnish Biobank Act came into effect (in September 2013) and start of FinnGen (August 2017), were collected based on study-specific consents and later transferred to the Finnish biobanks after approval by Fimea (Finnish Medicines Agency), the National Supervisory Authority for Welfare and Health. Recruitment protocols followed the biobank protocols approved by Fimea. The Coordinating Ethics Committee of the Hospital District of Helsinki and Uusimaa (HUS) statement number for the FinnGen study is HUS/990/2017.

The FinnGen study is approved by Finnish Institute for Health and Welfare (permits THL/2031/6.02.00/2017, THL/1101/5.05.00/2017, THL/341/6.02.00/2018, THL/2222/6.02.00/2018, THL/283/6.02.00/2019, THL/1721/5.05.00/2019 and THL/1524/5.05.00/2020), Digital and population data service agency (permits VRK43431/2017-3, VRK/6909/2018-3 and VRK/4415/2019-3), the Social Insurance Institution (KELA; permits KELA 58/522/2017, KELA 131/522/2018, KELA 70/522/2019, KELA 98/522/2019, KELA 134/522/2019, KELA 138/522/2019, KELA 2/522/2020 and KELA 16/522/2020), Findata permits (THL/2364/14.02/2020, THL/4055/14.06.00/2020, THL/3433/14.06.00/2020, THL/4432/14.06/2020, THL/5189/14.06/2020, THL/5894/14.06.00/2020, THL/6619/14.06.00/2020, THL/209/14.06.00/2021, THL/688/14.06.00/2021, THL/1284/14.06.00/2021, THL/1965/14.06.00/2021, THL/5546/14.02.00/2020, THL/2658/14.06.00/2021 and THL/4235/14.06.00/2021), Statistics Finland (permits TK-53-1041-17 and TK/143/07.03.00/2020 (earlier TK-53-90-20), TK/1735/07.03.00/2021 and TK/3112/07.03.00/2021) and Finnish Registry for Kidney Diseases permission/extract from the meeting minutes on 4 July 2019.

The Biobank Access Decisions for FinnGen samples and data utilized in FinnGen Data Freeze 10 include the following: THL Biobank (BB2017_55, BB2017_111, BB2018_19, BB2018_34, BB2018_67, BB2018_71, BB2019_7, BB2019_8, BB2019_26, BB2020_1 and BB2021_65), Finnish Red Cross Blood Service Biobank (7 December 2017), Helsinki Biobank (HUS/359/2017, HUS/248/2020 and HUS/150/2022: §§12, 13, 14, 15, 16, 17, 18 and 23), Auria Biobank (AB17-5154 and amendment 1 (17 August 2020); BB_2021-0140, BB_2021-0156 (26 August 2021 and 2 February 2022), BB_2021-0169, BB_2021-0179 and BB_2021-0161; AB20-5926 and amendment 1 (23 April 2020) and its modification (22 September 2021)), Biobank Borealis of Northern Finland (2017_1013, 2021_5010, 2021_5018, 2021_5015, 2021_5023, 2021_5017 and 2022_6001), Biobank of Eastern Finland (1186/2018 and amendments §§22/2020, 53/2021, 13/2022, 14/2022 and 15/2022), Finnish Clinical Biobank Tampere (MH0004 and amendments (21 February 2020 and 6 October 2020); §§8/2021, 9/2022, 10/2022, 12/2022, 20/2022, 21/2022, 22/2022 and 23/2022), Central Finland Biobank (1-2017), Terveystalo Biobank (STB 2018001 and amendment (25 August 2020)), Finnish Hematological Registry and Clinical Biobank (decision dated 18 June 2021) and Arctic Biobank (P0844: ARC_2021_1001).

### Study population

In the current study, we included samples from 425,483 individuals from Finland, sourced from FinnGen Data Freeze 10 (https://www.finngen.fi/en)^21^. This biobank study includes samples from hospital biobanks, alongside prospective epidemiological and disease-based cohorts. Using the unique national personal identification numbers, the data were interconnected with national registries including hospital discharge records (accessible from 1968), death records (from 1969), cancer registries (from 1953) and drug purchase records (from 1995). Registry information was accessible up to 31 December 2021.

### Trial selection

As the currently largest trial emulation effort, the RCT-DUPLICATE project^6,26^ has been emulating numerous RCTs in US-American insurance claims datasets, the goal of which was to assess the utility of the obtained RWE for regulatory decision-making.

We sought to identify four RCTs that have been previously replicated by RCT-DUPLICATE and were feasible to be successfully emulated in our RWD dataset. By the time of initiation of our project, findings of the first ten trial emulations were published by the RCT-DUPLICATE project^26^. The evaluation criteria deciding upon the feasibility of a RCT replication included critical aspects of the trial emulation protocol, such as the primary outcomes, eligibility criteria, treatment strategies, allowing for only minor deviations if features were not available in our data source (Supplementary Tables 2–3). The RCT was seen as closely emulated when the emulation of the comparator and outcome were at least moderate, and at least one of them was good, as described in the meta-analysis of RCT-DUPLICATE data^48^.

### Trial emulation design and analysis

Based on RCT-DUPLICATE’s trial emulation efforts, we developed the protocols for the emulations of four trials (Empareg, Tecos, Aristotle and Rocket). Closely following the original trial protocols, we emulated an observational data protocol for each trial, including the eligibility criteria, treatment strategies, assignment procedures, follow-up periods, primary outcomes, causal contrasts and an analysis plan^4^.

Different sets of eligibility criteria required fulfillment within distinct timeframes before therapy initiation. Flowcharts of cohort formations can be found in Supplementary Tables 5–8 and Supplementary Figs. 8–10.

The treatment strategies included new users of either the drug of interest or the comparator drug, starting from the date the newer drug received marketing authorization in Finland. For the two placebo-controlled trials, Empareg and Tecos, we selected an active comparator as a proxy for placebo regarding cardiovascular effects, similar to RCT-DUPLICATE. This is due to the fact that confounding bias may become especially serious when active user groups are compared to nonuser groups, as nonuser comparator groups considerably differ from actively treated patients in ways that are poorly captured in observational datasets^49,50^.

As a proxy for placebo, DPP4 inhibitors were chosen for Empareg, and second-generation sulfonylureas were chosen for Tecos, given they are likewise antidiabetic treatments commonly prescribed interchangeably with the treatments of interest and are known not to have any causal effect on cardiovascular outcomes based on current evidence^23,51–53^.

As the assignment procedures in observational studies are never at random, an adjustment for confounding variables is required to satisfy the exchangeability assumption. We selected sets of >25 confounding variables, measured within 6 months before drug initiation, reflecting demographics, comorbidities, comedications and cardiovascular procedures. We adopted 1:1 PS nearest-neighbor matching with a caliper of 0.1 or 0.01 on the PS scale, depending on the initial overlap^54,55^. PS matching statistics and details on covariate balance for all trial emulations can be found in Supplementary Tables 9–12.

Follow-up started at the first purchase of either of the defined therapeutics and ended with the occurrence of a primary outcome event, death, discontinuation or switch to a comparator or end of registry information, whichever occurs first. The time point of a discontinuation of therapy was calculated based on the number of packages purchased by the patient multiplied by the package size.

The primary outcome for Empareg and Tecos was 3P-MACE, and for Aristotle and Rocket, a composite endpoint of stroke and systemic embolism, adapted from the definition used in the corresponding trials.

In our analysis, we used an ‘on-treatment’ approach, attempting to replicate an intention-to-treat estimate derived from the RCT with particularly high treatment compliance. HR and 95% CI were estimated in PS-matched cohorts using the Cox proportional hazard models. We defined ‘estimate agreement’ as the emulation estimate being within the 95% CI for the RCT estimate.

### PGS generation

We computed genome-wide PGS for 20 traits (Supplementary Table 13) using the PGS-continuous shrinkage (CS) priors method, used in the PRS-CS tool (version 14 May 2024; https://github.com/getian107/PRScs)^56^. The input weights were derived from available summary statistics sourced from external genome-wide association studies (GWAS) data pertaining to the 20 traits. Variants were restricted to those present in the HapMap 3 reference panel^57^. To ensure comparability, PGS were standardized (mean = 0; s.d. = 1) in the whole FinnGen population. Detailed information regarding the summary statistics can be found in the Supplementary Information.

### PGS analysis of cohorts

We investigated genetic differences between the treated and control groups at three different stages of the emulation process and how they change with increased confounder adjustment.

For each PGS, we calculated the SMD between the treated and control groups using logistic regression and determined its significance based on a Bonferroni-corrected *P* value threshold (2.5 × 10^−3^).

In the first stage, we looked at a plain observational setting that best reflected the original RCT question. Therefore, as Empareg and Tecos are both placebo-controlled trials, we defined the plain observational setting as initiators of the treatment versus noninitiators. Because Aristotle and Rocket are both active-comparator trials, the plain observational setting was defined as initiators of the treatment versus initiators of the active comparator. In the second stage, we looked at the cohorts after applying the eligibility criteria. In the third, we considered the PS-matched cohorts.

### Simulations

To show that correcting on an imperfect proxy of the confounder can result in bias in effect size estimates, we carried out simulation experiments under the causal model shown in Fig. 3a. We first generated PGS as a random variable following the standard normal distribution *n*(0,1), and the rest of the variables were subsequently created as

$$C={\mathrm{rPGS}}+\sqrt{(1-{r}^{2})}{\varepsilon }_{C},$$

$$X={b}_{{CX}}C+\sqrt{(1-{b}_{{CX}}^{2})}{\varepsilon }_{X},$$

and $$Y=X+{b}_{{CY}}C$$, where $${\varepsilon }_{C},{\varepsilon }_{X} \sim n(0,1)$$.

The variables were simulated as such so that the variance of PGS, *C* and *X* were all 1, and the expected effect of *X* on *Y* is 1. Under this model, we could change the correlation between *C* and PGS simply by varying *r*, and the effect of the confounding factor *C* on *X* and *Y* by varying *b*_*CX*_ and *b*_*CY*_. Under each condition, we measured the observed effect of *X* on *Y*, conditioned on PGS, which was an imperfect proxy of *C*, through linear regression ${\rm{lm}}(Y \sim X+{\mathrm{PGS}})$. We denoted estimated bias as the observed regression coefficient −1, which is the expected underlying effect of *X* on *Y*.

We also wanted to demonstrate that even under a fixed correlation coefficient between *C* and PGS, the extent of bias in the observed *X* on *Y* effect can still vary due to additional components contributing to only PGS and *X*, *Y*, but not *C.* We further carried out simulations under a different causal model shown in Supplementary Fig. 6a, where PGS and confounder *C* are correlated due to a common underlying causal factor ${G}^{* }$. Meanwhile, an extra component *G*′ contributes only to PGS but not to *C*. Under this model, we first generated the shared causal factor ${G}^{* }$ and the PGS unique causal factor *G*′ independently following the standard normal distribution *n*(0,1) and other variables as below:

$${\mathrm{PGS}}={b}_{{G}^{*}\,{\rm{PGS}}}\,{G}^{* }+\sqrt{(1-{b}_{{G}^{*}\,{\rm{PGS}}}^{2})}G^{\prime}$$

$C={b}_{{G}^{*}C}\,{G}^{* }+\sqrt{(1-{b}_{{G}^{* }C}^{2})}{\varepsilon }_{C}$, where ${b}_{{G}^{* }C}=\frac{r}{{b}_{{G}^{* }\,{\mathrm{PGS}}}}$ and *r* is the correlation coefficient between *C* and PGS. We fixed the contribution of ${G}^{* }$ on PGS as $\frac{{b}_{{G}^{* }{\mathrm{PGS}}}^{2}}{{\mathrm{Var}}({\mathrm{PGS}})}$ = 0.8 and *r*^*2*^ = 0.3 in this experiment.

Subsequently, we simulated *X* and *Y* as

$$X={b}_{G^{\prime} X}\,G^{\prime} +{b}_{{CX}}\,C+\sqrt{(1-{b}_{G^{\prime} X}^{2}-{b}_{{CX}}^{2})}{\varepsilon }_{C}\,{\rm{and}}\,{Y}=X+{b}_{G^{\prime} Y}\,G^{\prime} +{b}_{{CY}}\,C$$

The variables were simulated as such so that the variance of *G*′, G*, PGS, *C* and *X* were all 1, and the expected effect of *X* on *Y* is 1. In this experiment, for simplicity, we fixed the contribution of *C* on *X* and *Y* so that $\frac{{\mathrm{Var}}({b}_{{CX}}C)}{{\mathrm{Var}}(X)}=\frac{{\mathrm{Var}}({b}_{{CY}}C)}{{\mathrm{Var}}(Y)}=\,0.3$, and assumed that *G*′ has no effect on *Y*. Furthermore, as a proof of concept, we assumed that *G*′ has a negative effect on *X* (${b}_{G{\prime} X}^{2} < 0$) because in this case, we expect to see an increment in estimate bias when *G*′ contributes more to the variance of *X*. We looked at estimate bias from a same linear regression ${\rm{lm}}(Y \sim X+{\mathrm{PGS}})$ in respect of changes in $\frac{(1-{b}_{{G}^{* }{\mathrm{PGS}}}^{2})}{{\mathrm{Var}}({\mathrm{PGS}})}$ and $\frac{{b}_{G{\prime} X}^{2}}{{\mathrm{Var}}(X)}$.

### Genome-wide association studies

We used REGENIE (v2.2.4)^58^ to perform a GWAS of empagliflozin initiation in the whole population, including 426,775 samples (cases, 14,996; controls, 411,779), as well as after applying the eligibility criteria of the Empareg emulation, including 11,349 samples (cases, 4,630; controls, 6,719). Details on genotyping and imputation in FinnGen can be found in ref. ^21^.

### MR analysis

By using genetic variants as IVs, we used two-sample MR to investigate the confounding status of numerous variables in a trial emulation setting^18^. In our MR analysis, we only focused on the Empareg trial emulation. We examined the effect of the 20 traits used in the PGS analysis (sources of external summary statistics can be found in Supplementary Table 13) on the trial outcome, represented by summary statistics for CHD, as well as on receiving the empagliflozin treatment in the whole population and after applying the eligibility criteria, both represented by summary statistics from our GWASs. We performed the MR analysis using the inverse variance-weighted (IVW) method.

To obtain the independent IVs for each trait, we filtered for significant exposure-associated single-nucleotide polymorphisms (SNPs) (*P* < 5 × 10^−8^), performed linkage disequilibrium clumping (*r*^2^ < 0.001; clumping window = 10,000 kb) and excluded potential outcome-associated SNPs (defined as *P* < 5 × 10^−8^ with the outcome).

We identified the following three key steps in using MR to explore confounding: (1) MR of the potential confounder on treatment. Conducting an MR analysis to assess the causal effect of the proposed confounding trait on the treatment variable. If the MR analysis shows a significant association, it suggests the potential confounder is indeed related to the treatment. (2) MR of potential confounder on outcome. Performing a separate MR analysis to evaluate the causal effect of the proposed confounding trait on the trial outcome variable. If the MR analysis demonstrates a significant association, it indicates the potential confounder is also related to the outcome. (3) Interpretation. If both MR analyses (steps 1 and 2) show significant associations, it implies the proposed trait is very likely to be a true confounder that needs to be accounted for and addressed through statistical adjustment in the trial emulation to obtain widely unbiased average treatment effects. Expert knowledge is still required to assess the plausibility of the MR analyses.

### Statistical analysis of the outcome PGS within trial emulations

Analogously to the MR analysis, we selected the CHD PGS as outcome PGS for MACE and stroke PGS for the composite endpoint stroke/systemic embolism. We evaluated the effect of the outcome PGS on the primary outcome within each PS-matched cohort using Cox regression and adjusting for the treatment.

$$h({t|T},{\mathrm{PGS}})={h}_{0}(t)\times \exp ({\beta }_{1}\times T+{\beta }_{2}\times {\mathrm{PGS}}),$$

where $h(t)$ is the hazard at time *t*, ${h}_{0}(t)\,$ is the baseline hazard at time *t*, $T$ is the treatment group, ${\mathrm{PGS}}$ is the outcome PGS, and ${\beta }_{1}$ and ${\beta }_{2}$ are coefficients associated with the treatment group variable $T$ and ${\mathrm{PGS}}$, respectively.

Additionally, we predicted the outcome of PGS effects on the primary outcome in the full population using Cox regression. Survival times started at birth with follow-up until the occurrence of the primary outcome, death or end of registry information, whichever occurred first.

Furthermore, we determined the event rate of the primary outcome for each trial and investigated the event rates within individuals with the top 25% PGS. Based on that, we calculated the required sample sizes given the new event rates to reach the same statistical power. This was to assess the effect of PGS enrichment on sample sizes in clinical trials.

### Reporting summary

Further information on research design is available in the Nature Portfolio Reporting Summary linked to this article.

## Online content

Any methods, additional references, Nature Portfolio reporting summaries, source data, extended data, supplementary information, acknowledgements, peer review information; details of author contributions and competing interests; and statements of data and code availability are available at 10.1038/s41588-025-02229-8.

## Supplementary information

Supplementary Information Supplementary Figs. 1–10 and FinnGen author banner (consortium).

Reporting Summary

Supplementary Tables Supplementary Tables 1–13.

Supplementary Data 1 Supporting data to generate Supplementary Figs. 1–6.

## Source data

Source Data Figs. 1–6 Source data to generate Figs. 1–6.

## Data Availability

Access to individual-level sensitive health data, as mandated by National and European Regulation (GDPR), requires approval from national authorities for specific research projects and for researchers who are explicitly listed and approved. The health data referenced in this study were generated and provided by the National Health Register Authorities (Finnish Institute of Health and Welfare, Statistics Finland, KELA, Digital and Population Data Services Agency) and approved by either the respective authorities or the Finnish Data Authority, Findata, for use in the FinnGen project. As a result, we, the authors, are unable to grant access to individual-level data to third parties. However, researchers can apply for access to the health register data through the Finnish Data Authority, Findata (https://findata.fi/en/permits/), and for individual-level genotype data from Finnish biobanks through the Fingenious portal (https://site.fingenious.fi/en/), managed by the Finnish Biobank Cooperative FINBB (https://finbb.fi/en/). All Finnish biobanks can provide data for research projects under the scope of the Finnish Biobank Act, which includes research aimed at promoting health, understanding disease mechanisms or developing health and medical care products and practices. More information on accessing FinnGen data can be found at https://www.finngen.fi/en/access_results. A comprehensive list of FinnGen endpoints is available at https://www.finngen.fi/en/researchers/clinical-endpoints. Source data are provided with this paper.

## Code availability

The study used previously published analysis tools as described in the Methods. Additional code used for these analyses is available at Zenodo^59^ and can be found under https://github.com/dsgelab/trial_emulations_genetics.
