# Author Correction: Incorporating genetic data improves target trial emulations and informs the use of polygenic scores in randomized controlled trial design

**DOI:** 10.1038/s41588-025-02275-2

**Published:** 2025-06-27

**Authors:** Jakob German, Zhiyu Yang, Sarah Urbut, Pekka Vartiainen, FinnGen, Andrea Gana, Pradeep Natarajan, Elisabetta Patorno, Zoltan Kutalik, Anthony Philippakis, Andrea Ganna

**Affiliations:** 1 Institute for Molecular Medicine Finland (FIMM), University of Helsinki, Helsinki, Finland; 2 Eric and Wendy Schmidt Center, Broad Institute of MIT and Harvard, Cambridge, MA USA; 3 Division of Cardiovascular Medicine, Massachusetts General Hospital, Boston, MA USA; 4 Center for Genomic Medicine Massachusetts General Hospital, Boston, MA USA; 5 Cardiovascular Research Center, Massachusetts General Hospital, Boston, MA USA; 6 Pediatric Research Center, Helsinki University Hospital and University of Helsinki, Helsinki, Finland; 7 Program in Medical and Population Genetics, Broad Institute of MIT and Harvard, Cambridge, MA USA; 8 Personalized Medicine, Mass General Brigham, Boston, MA USA; 9 Department of Medicine, Harvard Medical School, Boston, MA USA; 10 Division of Pharmacoepidemiology and Pharmacoeconomics, Department of Medicine, Brigham and Women’s Hospital and Harvard Medical School, Boston, MA USA; 11 University Center for Primary Care and Public Health, Lausanne, Switzerland; 12 Department of Computational Biology, University of Lausanne, Lausanne, Switzerland; 13 Swiss Institute of Bioinformatics, Lausanne, Switzerland; 14 Broad Institute of MIT and Harvard, Cambridge, MA USA; 15 Analytic and Translational Genetics Unit, Massachusetts General Hospital, Harvard Medical School, Boston, MA USA

**Keywords:** Genetics research, Randomized controlled trials, Epidemiology

Correction to: *Nature Genetics* 10.1038/s41588-025-02229-8. Published online 18 June 2025.

In the version of this article originally published, the surname of Elisabetta Patorno was spelled incorrectly (Pattorno) and is now amended in the HTML and PDF versions of the article.

