# Supplementary material for: Incorporating genetic data improves target trial emulations and informs the use of polygenic scores in randomized controlled trial design
Source: Nat Genet. 2025 Jun 18;57(7):1620–7. doi: 10.1038/s41588-025-02229-8 (PMC12283355; doi:10.1038/s41588-025-02229-8)
Supplement: Supplementary file 2 — Reporting Summary [file 41588_2025_2229_MOESM2_ESM.pdf]

nature portfolio

Corresponding author(s): Andrea Ganna

Last updated by author(s): Apr 8, 2025

# Reporting Summary

Nature Portfolio wishes to improve the reproducibility of the work that we publish. This form provides structure for consistency and transparency in reporting. For further information on Nature Portfolio policies, see our Editorial Policies and the Editorial Policy Checklist.

## Statistics

For all statistical analyses, confirm that the following items are present in the figure legend, table legend, main text, or Methods section.

| n/a | Confirmed | |
|---|---|---|
| ☐ | ☒ | The exact sample size (*n*) for each experimental group/condition, given as a discrete number and unit of measurement |
| ☐ | ☒ | A statement on whether measurements were taken from distinct samples or whether the same sample was measured repeatedly |
| ☐ | ☒ | The statistical test(s) used AND whether they are one- or two-sided<br>*Only common tests should be described solely by name; describe more complex techniques in the Methods section.* |
| ☐ | ☒ | A description of all covariates tested |
| ☐ | ☒ | A description of any assumptions or corrections, such as tests of normality and adjustment for multiple comparisons |
| ☐ | ☒ | A full description of the statistical parameters including central tendency (e.g. means) or other basic estimates (e.g. regression coefficient) AND variation (e.g. standard deviation) or associated estimates of uncertainty (e.g. confidence intervals) |
| ☐ | ☒ | For null hypothesis testing, the test statistic (e.g. *F*, *t*, *r*) with confidence intervals, effect sizes, degrees of freedom and *P* value noted<br>*Give P values as exact values whenever suitable.* |
| ☒ | ☐ | For Bayesian analysis, information on the choice of priors and Markov chain Monte Carlo settings |
| ☒ | ☐ | For hierarchical and complex designs, identification of the appropriate level for tests and full reporting of outcomes |
| ☒ | ☐ | Estimates of effect sizes (e.g. Cohen's *d*, Pearson's *r*), indicating how they were calculated |

*Our web collection on statistics for biologists contains articles on many of the points above.*

## Software and code

Policy information about availability of computer code

| | |
|---|---|
| Data collection | No software was used for data collection. |
| Data analysis | All analysis steps and related tools are described in the Methods section. The software used and their versions are:<br>R v4.1<br>ICCI R package v1 https://github.com/dsgelab/ICCI<br>PRS-CS (version May 14, 2024) https://github.com/getian107/PRScs<br>REGENIE v2.2.4 doi: 10.1038/s41588-021-00870-7<br><br>Analysis code used to produce the results is available on GitHub at: https://github.com/dsgelab/trial_emulations_genetics |

For manuscripts utilizing custom algorithms or software that are central to the research but not yet described in published literature, software must be made available to editors and reviewers. We strongly encourage code deposition in a community repository (e.g. GitHub). See the Nature Portfolio guidelines for submitting code & software for further information.

# Data

Policy information about availability of data

All manuscripts must include a data availability statement. This statement should provide the following information, where applicable:

- Accession codes, unique identifiers, or web links for publicly available datasets
- A description of any restrictions on data availability
- For clinical datasets or third party data, please ensure that the statement adheres to our policy

Access to individual-level sensitive health data, as mandated by National and European regulations (GDPR), requires approval from national authorities for specific research projects and for researchers who are explicitly listed and approved. The health data referenced in this study was generated and provided by the National Health Register Authorities (Finnish Institute of Health and Welfare, Statistics Finland, KELA, Digital and Population Data Services Agency) and approved by either the respective authorities or the Finnish Data Authority, Findata, for use in the FinnGen project. As a result, we, the authors, are unable to grant access to individual-level data to third parties. However, researchers can apply for access to the health register data through the Finnish Data Authority, Findata (https://findata.fi/en/permits/), and for individual-level genotype data from Finnish biobanks through the Fingenious portal (https://site.fingenious.fi/en/), managed by the Finnish Biobank Cooperative FINBB (https://finbb.fi/en/). All Finnish biobanks can provide data for research projects under the scope of the Finnish Biobank Act, which includes research aimed at promoting health, understanding disease mechanisms, or developing health and medical care products and practices. More information on accessing FinnGen data can be found here: https://www.finngen.fi/en/access_results. A comprehensive list of FinnGen endpoints is available at: https://www.finngen.fi/en/researchers/clinical-endpoints.

# Research involving human participants, their data, or biological material

Policy information about studies with human participants or human data. See also policy information about sex, gender (identity/presentation), and sexual orientation and race, ethnicity and racism.

| | |
|---|---|
| Reporting on sex and gender | Sex was geneticallly determined in FinnGen. Related policy is specified in the FinnGen study (https://www.finngen.fi/en/researchers/data_available) |
| Reporting on race, ethnicity, or other socially relevant groupings | Related policy is specified in the FinnGen study (https://www.finngen.fi/en/researchers/data_available) |
| Population characteristics | Biobanks provide age, sex, height, weight, smoking and DNA sample collection date variables. In addition, information about the region of birth, moving abroad and the number of children from the Digital and population data services agency is included. Cohort data includes information about the biobank that has delivered the sample in question to FinnGen. Further, health register codes from six Finnish health registers are used to determine which individuals fall into each FinnGen endpoint. The full catalog of the FinnGen endpoints has been created to comprehensively cover the spectrum of medical diagnoses from all medical areas.<br><br>Related policy is specified in the FinnGen study (https://www.finngen.fi/en/researchers/data_available) |
| Recruitment | FinnGen is a biobank study, which means that the samples FinnGen uses have been collected by the Finnish biobanks and FinnGen has applied for permission to use the samples, as outlined in the Finnish Biobank Act.<br>The FinnGen cohort was deliberately structured to include a disproportionate number of individuals with diseases. This was achieved through a combination of pre-existing collections, characterised by a relatively high average participant age, and targeted recruitment from specific hospital clinics to capture less common diseases and those associated with advanced age, which are typically underrepresented in most population studies. This deliberate enrichment strategy offers FinnGen distinct advantages for uncovering genetic factors related to diseases.<br><br>Please find the relevant information here: https://www.finngen.fi/en/node/1985 |
| Ethics oversight | Patients and control subjects in FinnGen provided informed consent for biobank research, based on the Finnish Biobank Act. Alternatively, separate research cohorts, collected prior the Finnish Biobank Act came into effect (in September 2013) and start of FinnGen (August 2017), were collected based on study-specific consents and later transferred to the Finnish biobanks after approval by Fimea (Finnish Medicines Agency), the National Supervisory Authority for Welfare and Health. Recruitment protocols followed the biobank protocols approved by Fimea. The Coordinating Ethics Committee of the Hospital District of Helsinki and Uusimaa (HUS) statement number for the FinnGen study is Nr HUS/990/2017.<br><br>The FinnGen study is approved by Finnish Institute for Health and Welfare (permit numbers: THL/2031/6.02.00/2017, THL/1101/5.05.00/2017, THL/341/6.02.00/2018, THL/2222/6.02.00/2018, THL/283/6.02.00/2019, THL/1721/5.05.00/2019 and THL/1524/5.05.00/2020), Digital and population data service agency (permit numbers: VRK43431/2017-3, VRK/6909/2018-3, VRK/4415/2019-3), the Social Insurance Institution (permit numbers: KELA 58/522/2017, KELA 131/522/2018, KELA 70/522/2019, KELA 98/522/2019, KELA 134/522/2019, KELA 138/522/2019, KELA 2/522/2020, KELA 16/522/2020), Findata permit numbers THL/2364/14.02/2020, THL/4055/14.06.00/2020, THL/3433/14.06.00/2020, THL/4432/14.06/2020, THL/5189/14.06/2020, THL/5894/14.06.00/2020, THL/6619/14.06.00/2020, THL/209/14.06.00/2021, THL/688/14.06.00/2021, THL/1284/14.06.00/2021, THL/1965/14.06.00/2021, THL/5546/14.02.00/2020, THL/2658/14.06.00/2021, THL/4235/14.06.00/2021, Statistics Finland (permit numbers: TK-53-1041-17 and TK/143/07.03.00/2020 (earlier TK-53-90-20) TK/1735/07.03.00/2021, TK/3112/07.03.00/2021) and Finnish Registry for Kidney Diseases permission/extract from the meeting minutes on 4th July 2019.<br><br>The Biobank Access Decisions for FinnGen samples and data utilized in FinnGen Data Freeze 10 include: THL Biobank |

BB2017_55, BB2017_111, BB2018_19, BB_2018_34, BB_2018_67, BB2018_71, BB2019_7, BB2019_8, BB2019_26, BB2020_1, BB2021_65, Finnish Red Cross Blood Service Biobank 7.12.2017, Helsinki Biobank HUS/359/2017, HUS/248/2020, HUS/150/2022 § 12, §13, §14, §15, §16, §17, §18, and §23, Auria Biobank AB17-5154 and amendment #1 (August 17 2020) and amendments BB_2021-0140, BB_2021-0156 (August 26 2021, Feb 2 2022), BB_2021-0169, BB_2021-0179, BB_2021-0161, AB20-5926 and amendment #1 (April 23 2020)and it´s modification (Sep 22 2021), Biobank Borealis of Northern Finland_2017_1013, 2021_5010, 2021_5018, 2021_5015, 2021_5023, 2021_5017, 2022_6001, Biobank of Eastern Finland 1186/2018 and amendment 22 § /2020, 53§/2021, 13§/2022, 14§/2022, 15§/2022, Finnish Clinical Biobank Tampere MH0004 and amendments (21.02.2020 & 06.10.2020), §8/2021, §9/2022, §10/2022, §12/2022, §20/2022, §21/2022, §22/2022, §23/2022, Central Finland Biobank 1-2017, and Terveystalo Biobank STB 2018001 and amendment 25th Aug 2020, Finnish Hematological Registry and Clinical Biobank decision 18th June 2021, Arctic biobank P0844: ARC_2021_1001.

Note that full information on the approval of the study protocol must also be provided in the manuscript.

# Field-specific reporting

Please select the one below that is the best fit for your research. If you are not sure, read the appropriate sections before making your selection.

☒ Life sciences ☐ Behavioural & social sciences ☐ Ecological, evolutionary & environmental sciences

For a reference copy of the document with all sections, see nature.com/documents/nr-reporting-summary-flat.pdf

# Life sciences study design

All studies must disclose on these points even when the disclosure is negative.

| | |
|---|---|
| Sample size | 425,483 individuals of FinnGen (release 10)<br>Sample size was determined based on the number of individuals for which genetic and phenotypic data were available in the study. No prior power calculations were performed to determine the required sample size. |
| Data exclusions | No individuals (from the total sample size) were excluded from the analysis.<br>Eligibility criteria for each trial emulation are based on original clinical trial protocols and are described in the Methods section and in the Supplementary Material. |
| Replication | Replication was not directly applicable to this study because we used a trial emulation approach, where we simulated the design and analysis of four major cardiometabolic RCTs using real-world data from FinnGen. Although we replicated the original trial protocols, including outcomes and eligibility criteria, the study aimed to emulate the trials rather than perform a traditional replication. This allowed us to investigate the role of genetic data in improving trial design and adjusting for confounders without repeating the trials themselves. |
| Randomization | This is an observational study. Randomization was emulated using the trial emulation framework. |
| Blinding | This is an observational study. Blinding was not applicable. |

# Reporting for specific materials, systems and methods

We require information from authors about some types of materials, experimental systems and methods used in many studies. Here, indicate whether each material, system or method listed is relevant to your study. If you are not sure if a list item applies to your research, read the appropriate section before selecting a response.

## Materials & experimental systems

| n/a | Involved in the study | |
|---|---|---|
| ☒ | ☐ | Antibodies |
| ☒ | ☐ | Eukaryotic cell lines |
| ☒ | ☐ | Palaeontology and archaeology |
| ☒ | ☐ | Animals and other organisms |
| ☒ | ☐ | Clinical data |
| ☒ | ☐ | Dual use research of concern |
| ☒ | ☐ | Plants |

## Methods

| n/a | Involved in the study | |
|---|---|---|
| ☒ | ☐ | ChIP-seq |
| ☒ | ☐ | Flow cytometry |
| ☒ | ☐ | MRI-based neuroimaging |

## Plants

| | |
|---|---|
| Seed stocks | *Report on the source of all seed stocks or other plant material used. If applicable, state the seed stock centre and catalogue number. If plant specimens were collected from the field, describe the collection location, date and sampling procedures.* |
| Novel plant genotypes | *Describe the methods by which all novel plant genotypes were produced. This includes those generated by transgenic approaches, gene editing, chemical/radiation-based mutagenesis and hybridization. For transgenic lines, describe the transformation method, the number of independent lines analyzed and the generation upon which experiments were performed. For gene-edited lines, describe the editor used, the endogenous sequence targeted for editing, the targeting guide RNA sequence (if applicable) and how the editor was applied.* |
| Authentication | *Describe any authentication procedures for each seed stock used or novel genotype generated. Describe any experiments used to assess the effect of a mutation and, where applicable, how potential secondary effects (e.g. second site T-DNA insertions, mosiacism, off-target gene editing) were examined.* |
